# Three-dimensional alkaline earth metal–organic framework poly[[μ-aqua-aqua­bis­(μ_3_-carba­moyl­cyano­nitro­somethanido)barium] monohydrate] and its thermal decomposition

**DOI:** 10.1107/S2056989024008375

**Published:** 2024-08-30

**Authors:** Kostiantyn V. Domasevitch, Ganna A. Senchyk, Vira V. Ponomarova, Andrey B. Lysenko, Harald Krautscheid

**Affiliations:** a Inorganic Chemistry Department National Taras Shevchenko University of Kyiv Volodymyrska Str 64/13 01601 Kyiv Ukraine; b Institute of Inorganic Chemistry, Leipzig University, Johannisallee 29, D-04103 Leipzig, Germany; Universität Greifswald, Germany

**Keywords:** crystal structure, carbamoyl­cyano­nitro­somethanide, nitroso-ligands, alkaline earth MOF materials, barium

## Abstract

In the structure of the title salt, {[Ba(μ_3_-C_3_H_2_N_3_O_2_)_2_(μ-H_2_O)(H_2_O)]·H_2_O}_*n*_, the barium ion and all three oxygen atoms of the water mol­ecules reside on a mirror plane. The hydrogen atoms of the bridging water and the solvate water mol­ecules are arranged across a mirror plane whereas all atoms of the monodentate aqua ligand are situated on this mirror plane. The distorted ninefold coord­ination of the Ba ions is completed with four nitroso-, two carbonyl- and three aqua-O atoms at the distances of 2.763 (3)–2.961 (4) Å and it is best described as tricapped trigonal prism. The three-dimensional framework structure is formed by face-sharing of the trigonal prisms, *via* μ-nitroso- and μ-aqua-O atoms, and also by the bridging coordination of the anions *via* carbonyl-O atoms occupying two out of the three cap positions. The solvate water mol­ecules populate the crystal channels and facilitate a set of four directional hydrogen bonds. The principal Ba–carbamoyl­cyano­nitro­somethanido linkage reveals a rare example of the inherently polar binodal six- and three-coordinated bipartite topology (three-letter notation **sit**). It suggests that small resonance-stabilized cyano­nitroso anions can be utilized as bridging ligands for the supra­molecular synthesis of MOF solids. Such an outcome may be anti­cipated for a broader range of hard Lewis acidic alkaline earth metal ions, which perfectly match the coordination preferences of highly nucleophilic nitroso-O atoms. Thermal analysis reveals two-stage dehydration of the title compound (383 and 473 K) followed by decomposition with release of CO_2_, HCN and H_2_O at 558 K.

## Abstract 2

A new alkaline earth metal three-dimensional polar MOF structure in barium carbamoyl­cyano­nitro­somethanide is governed by bridging coordination of the nitroso- and aqua O-atoms, which support a face-sharing connection of coordination polyhedra.

## 1. Chemical context

Alkaline earth metal-based framework coordination polymers offer significant advantages in the synthesis of functional solids for a range of applications, including gas storage and separation, proton conductivity, construction of optoelectronic devices and development of catalytic systems (Banerjee *et al.*, 2016 ▸; Király *et al.*, 2023 ▸). However, this class of materials remains relatively scarcely investigated. Designing their structures following the principles of supra­molecular synthesis and crystal engineering faces appreciable difficulties. This goes back to a lack of well-established and predictable coordination geometries, variable coordination numbers adopted by alkaline earth metal ions and a general trend for the coordination of solvent (aqua) mol­ecules. The latter, as terminal ligands, prevent the polymerization and formation of extended framework structures (Zang *et al.*, 2021 ▸).

It is not surprising that the library of organic linkers for the construction of these materials is essentially restricted to the main types of hard Lewis bases, such as carboxyl­ates (Banerjee & Parise, 2011 ▸), phospho­nates and sulfonates (Côté & Shimizu, 2003 ▸). All of them feature the availability of multiple donor atoms to fill the relatively capacious coordination spheres of the alkaline earth metal ions (Gagné & Hawthorne, 2016 ▸) and this is in line with the need to improve the predictability of coordination geometries with a larger number of donor atoms as well as to control the incorporation of terminal ligands. Herein, we report the engineering of a coordination framework with small resonance-stabilized carbamoyl­cyano­nitro­somethanide anions [(ONC(CN)—CONH_2_)^−^, (ccnm)^−^], which are employed as a new kind of linker in the context of alkaline earth metal-based three-dimensional materials. Such species may be well applied to the construction of framework solids, while exploiting specific preferences of the three present functional groups as hydrogen-bond acceptors (Turner *et al.*, 2011 ▸). For example, double N—H⋯O bonding of the nitroso-O atoms in a series of ammonium salts is a particularly reliable supra­molecular feature for extended structures with tuneable metrics and dimensionalities (Domasevitch *et al.*, 2023 ▸). In a similar fashion, when combined with the alkaline earth metal cations, the highly nucleophilic and sterically accessible nitroso-O atoms of (ccnm)^−^ could establish a suite of short-distance *M*—O—*M* bridges (Arulsamy *et al.*, 1999 ▸) and thus govern a predictable fusion of the coordination polyhedra. One can suppose that the demands for symmetrical electron distribution of the alkaline earth metal ions, which is the case when the bond valence vectors drawn from a central atom to its ligands will sum to zero (Müller *et al.*, 2003 ▸), could be best fulfilled by an identical face (edge) sharing at the opposite sides of the polyhedron. In total, this is a prerequisite of polymerization to yield an infinite connection of coordination polyhedra, which may be relatively controllable even in the case of very high coordination numbers. With this in mind, we now describe the synthesis and structure of the title salt, *poly*[[(μ-aqua)(aqua­barium)-bis-μ_3_-(carbamoyl­cyano­nitro­somethanido)] monohydrate] (**1**), which underlines the utility of bridging nitroso anions as linkers for the supra­molecular synthesis of a series of MOF solids with (potentially) various alkaline earth metal ions.
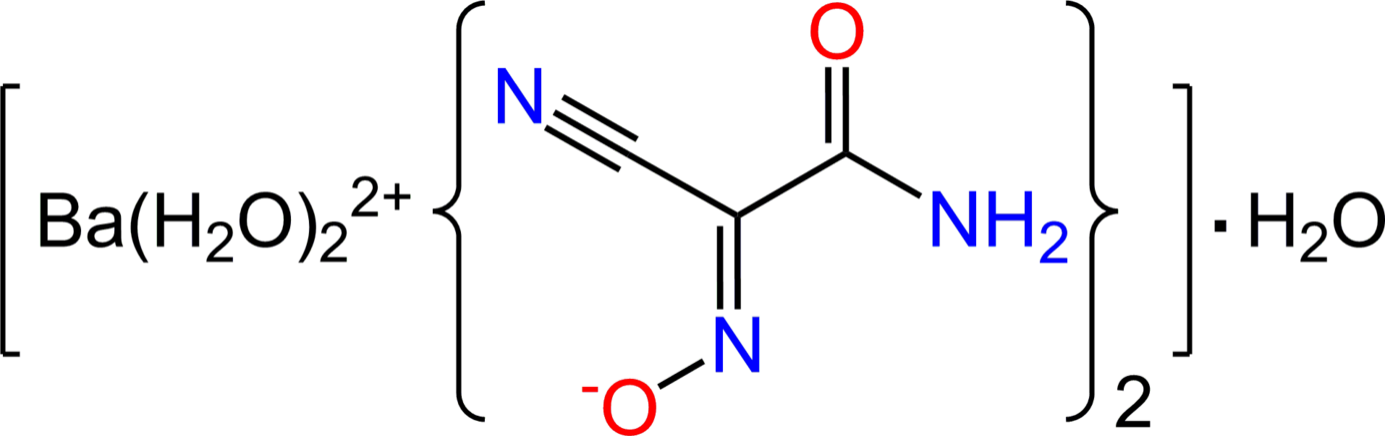


## 2. Structural commentary

**Figure 1:**
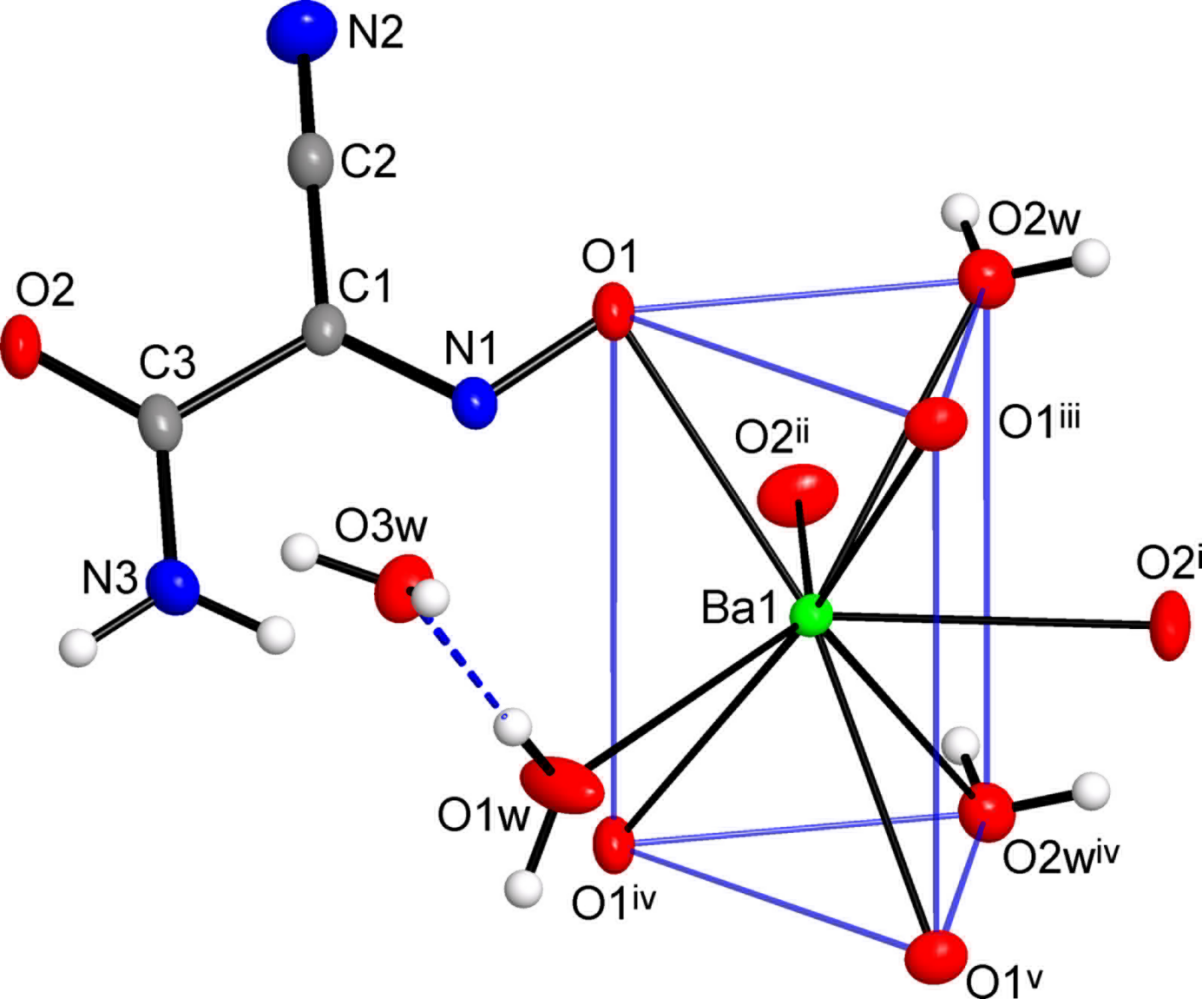
The mol­ecular structure of the title compound showing the ninefold environment of the Ba ion and displacement ellipsoids drawn at the 40% probability level. Blue lines indicate the trigonal–prismatic coordination core with three additional O atoms situated in the capped positions. Symmetry codes: (i) *x* − 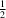, −*y* + 1, *z* − 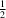; (ii) −*x* + 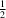, −*y* + 1, *z* − 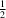; (iii) −*x*, *y*, *z*; (iv) *x*, *y* + 1, *z*; (v) −*x*, *y* + 1, *z*.

The mol­ecular structure of the title compound is shown in Fig. 1 ▸, with the asymmetric unit comprising the barium ion situated on a mirror plane, one organic ligand in a general position and three halves of water mol­ecules, of which one lies within the mirror plane and two others are oriented across the mirror plane.

The characteristic high ninefold coordination of the barium ion [BaO_9_] is completed with four nitroso-, two carbonyl- and three aqua-O atoms at distances of 2.763 (3)–2.961 (4) Å [mean 2.840 (5) Å, Table 1 ▸]. These values are fully consistent with the Ba—O separations for nine-coordinate environments in metal–organic structures (mean 2.860 Å; Gagné & Hawthorne, 2016 ▸) and also match the sum of the corresponding ionic radii of 2.87 Å (Shannon, 1976 ▸). In spite of the nearly uniform bond lengths, the environment is somewhat uneven, since the Ba ion is shifted by 0.390 (2) Å from the O_9_ group centroid toward the O1*W* position. This polar arrangement may be best assessed with a formalism of the bond-valence model (Brown, 2009 ▸). The bond-valence sum (BVS) for Ba1 is 2.04 and that is in good agreement with the theory. However, the vectorial sum of the bond-valence vectors corresponding to the individual bonded atoms represents a perceptible residual vector of 0.26 v.u., compared to a value of zero expected for alkaline earth metal ions (Harvey *et al.*, 2006 ▸). This distorted polyhedral geometry of the metal ion is nearly inter­mediate between the two most typical and very similar configurations of tricapped trigonal prism and capped square anti­prism, with the corresponding shape measures of 1.673 and 1.927, respectively (Ruiz-Martínez *et al.*, 2008 ▸). Therefore the attribution of the former configuration is essentially nominal, while facilitating further discussion of the three-dimensional connectivity. The vertices of the trigonal prism are occupied by four nitroso-O atoms of four anions and two O-atoms of the aqua ligands, whereas the three cap positions belong to two carbonyl-O atoms and the O atom of the terminal aqua ligand (Fig. 1 ▸).

The geometry parameters of the (ccnm)^−^ anion suggest a highly conjugated structure, which is common to a series of comparable small cyano anions (Turner *et al.*, 2011 ▸). The nitroso­cyano­methanide O1/N1/C1/C2/N2 and amide C3/N3/O2 fragments sustain a small dihedral angle of 8.3 (5)°, while the central methanide core itself is strictly planar (Fig. 1 ▸), as evidenced by the sum of the bond angles around C1 [359.8 (4)°]. The delocalization of the π-electron density in the (nccm)^−^ anions is indicated also by identical separations within the C N O fragment, which are C1—N1 = 1.313 (5) and N1—O1 = 1.313 (4) Å. This is contrary to the neutral H(ccnm) molecule that adopts the structure of a partially conjugated oxime C=N—OH, in which the two principal bond lengths are clearly distinct [1.288 (2) and 1.345 (2) Å, respectively, for H(ccnm)-3,4-di­methyl­pyrazole (1/1); Domasevitch *et al.*, 2024 ▸]. The present *trans–anti* configuration of the nitroso group with respect to the carbonyl group is the most typical for crystal structures of [ONC(CN)—CO*R*]^−^ salts (Domasevitch *et al.*, 1998 ▸; Ponomareva *et al.*, 1997 ▸), while the *syn* configuration of (ccnm)^−^ anions has only been detected spectroscopically in solution (Janikowski *et al.*, 2013 ▸).

## 3. Supra­molecular features

**Figure 2:**
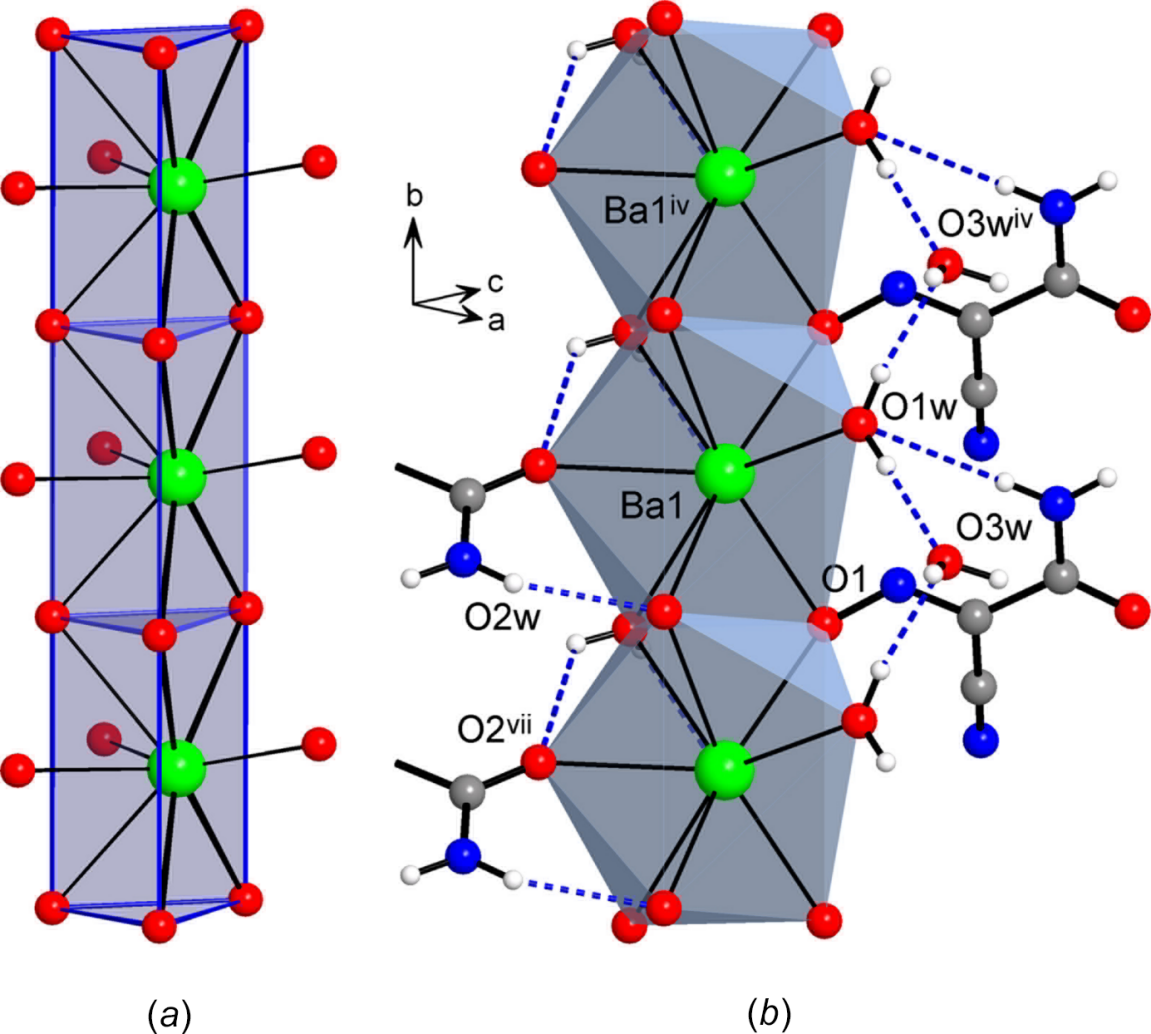
(*a*) Face-sharing connection of the coordination polyhedra through the triangular faces of the trigonal prisms; thick bonds indicate the primary Ba—O(nitroso) linkage. (*b*) The polyhedral chain accommodating organic anions and aqua ligands with a set of hydrogen-bond inter­actions represented by dotted blue lines. Symmetry codes: (iv) *x*, *y* + 1, *z*; (vii) −*x* + 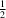, −*y*, *z* − 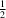.

**Figure 3:**
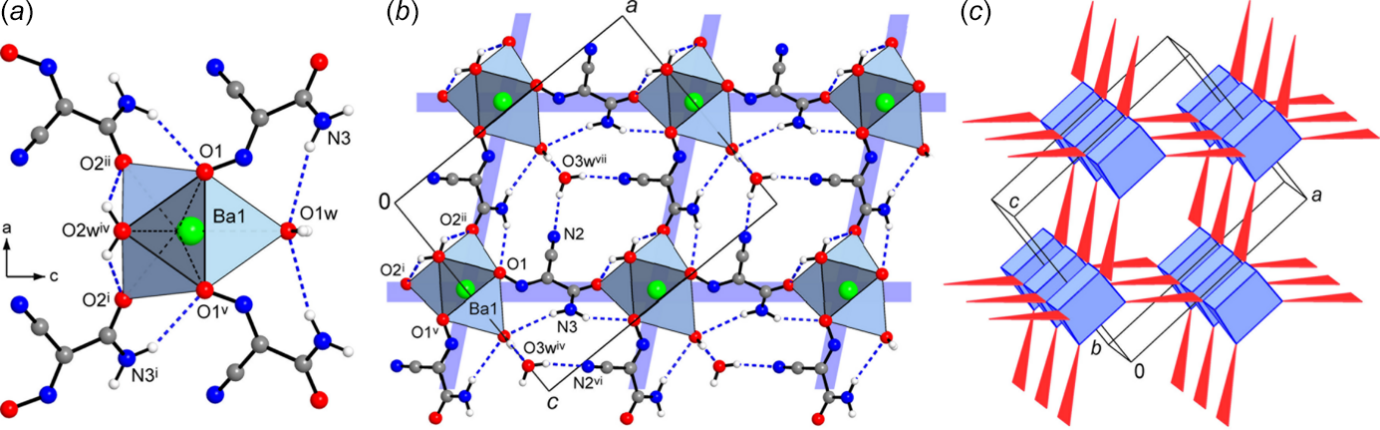
(*a*) Situation of four (ccnm)^−^ anions around the polyhedral stack, which is orthogonal to the drawing plane. (*b*) View of the framework down the *b*-axis direction, showing the principal coordination and hydrogen-bond inter­actions. (*c*) Topology of the metal–anion connectivity, in the form of a binodal heterocoordinated net with trigonal–prismatic [Ba^2+^] and trigonal [μ_3_-(ccnm)^−^] nodes, which are indicated in blue and red, respectively. Symmetry codes: (i) *x* − 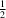, −*y* + 1, *z* − 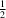; (ii) −*x* + 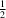, −*y* + 1, *z* − 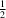; (iv) *x*, *y* + 1, *z*; (v) −*x*, *y* + 1, *z*; (vi) −*x* + 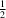, −*y* + 1, *z* + 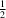; (vii) −*x* + 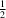, −*y*, *z* − 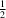.

The title compound adopts a three-dimensional framework structure with eight-connected Ba nodes, μ_3_-(ccnm)^−^ and μ-H_2_O links, found in a 1:2:1 proportion. The linkage is readily comprehensible when considering a sequence of face-sharing [BaO_9_] polyhedra, as simpler one-dimensional subconnectivities. The identical by symmetry triangular faces of the trigonal prisms are sustained with two nitroso- and one aqua-O atoms, each of which is bridging between the Ba ions of two translation-related fused polyhedra (Fig. 2 ▸). The Ba1⋯Ba1^iv^ separation of 4.4102 (7) Å [symmetry code: (iv) *x*, *y* + 1, *z*] corresponds to the *b*-axis parameter of the unit cell. In this way the trigonal prisms are stacked to yield linear chains along the *b*-axis direction. The (ccnm)^−^ groups, anchored to the polyhedral chains *via* nitroso-O atoms, provide the connection to adjacent chains *via* their carbonyl-O atoms, which occupy two out of the three cap positions at the Ba ions (Fig. 3 ▸). The remaining cap position hosts a terminal aqua ligand and therefore it does not influence the framework topology. One can formalize the principal connectivity when excluding the auxiliary μ-H_2_O links and consider the Ba ions and μ_3_-(ccnm)^−^ unit as the points of a binodal heterocoordinated topological net (Fig. 3 ▸*c*). In this way, a six- and three-connected framework is found with a point symbol {4.6^2^}_2_{4^2^.6^10^.8^3^} (three-letter notation **sit**; Blatov *et al.*, 2010 ▸). This topology is inherently polar. It represents a bipartite connection of trigonal–prismatic and trigonal nodes and it is relatively rarely associated with the crystal chemistry of metal–organic frameworks (Li *et al.*, 2014 ▸).

**Figure 4:**
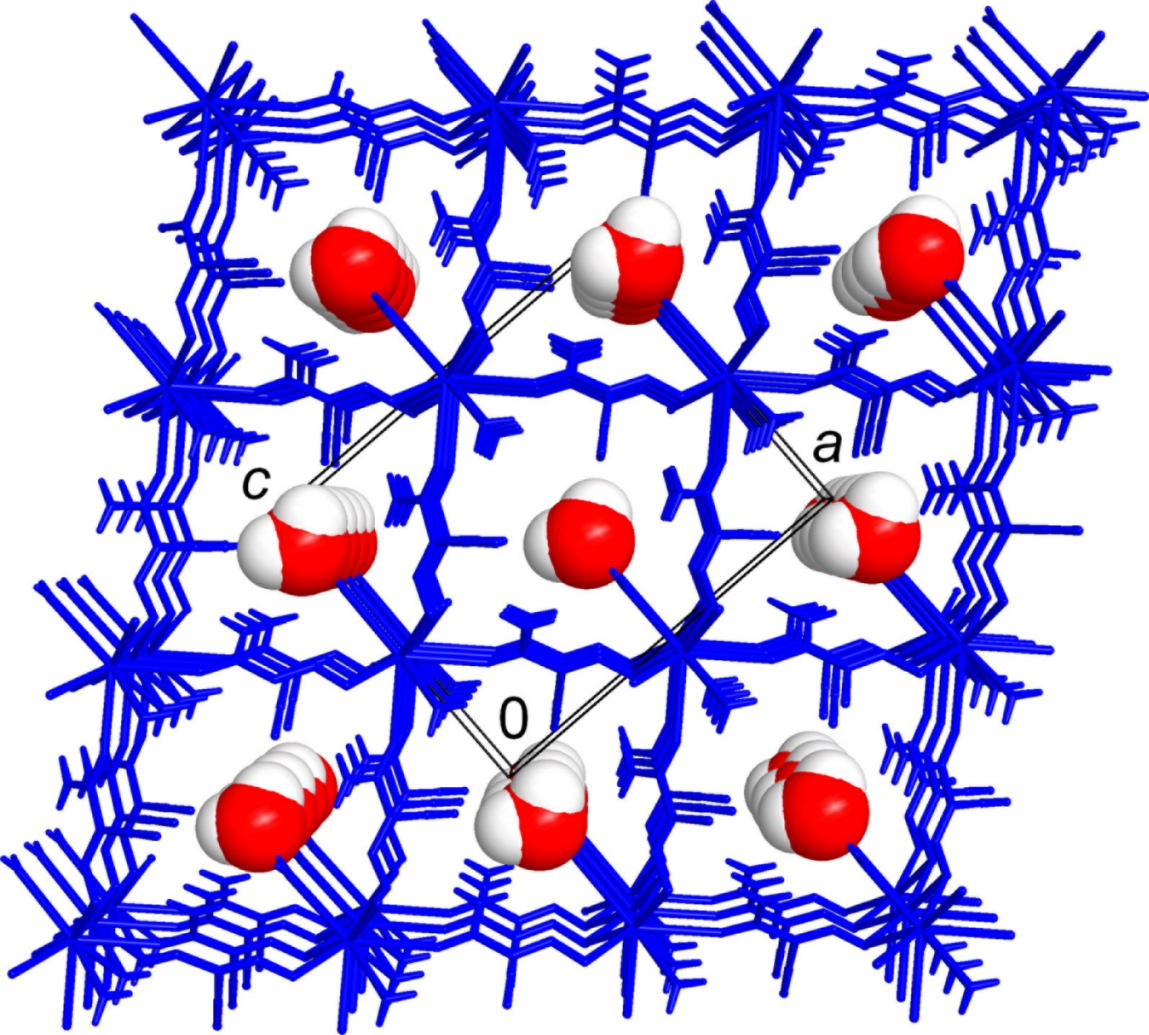
Perspective projection of the structure viewed down the *b* axis, which features small channels populated by solvate water mol­ecules.

The overall framework is relatively dense leaving only small channels with free volume accounting for 50 Å^3^ per unit cell or 7.4% of the crystal volume. These channels, running down the *b*-axis direction, are populated by solvate water mol­ecules (Fig. 4 ▸). The latter are important for extensive and relatively strong hydrogen bonding, which is superior to the strengths of other hydrogen-bond inter­actions in the structure. In particular, the solvate water mol­ecules reside in comfortable nearly tetra­hedral environments of two donors and two acceptors and afford four highly directional inter­actions, with the angles at the H atoms of 161 (5)–169 (9)° (Table 2 ▸). These mol­ecules are accommodated at the above polyhedral chains, while accepting a pair of O—H⋯O bonds from the terminal aqua ligands of the adjacent polyhedra [O⋯O = 2.782 (7) and 2.877 (7) Å] and extending the linkage to the pair of symmetry-equivalent nitrile-N acceptors [O3*W*⋯N2^viii^ = 2.933 (5) Å; symmetry code: (viii) −*x* + 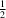, −*y*, *z* + 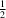] (Figs. 2 ▸, 3 ▸). Other possible hydrogen bonds involve the acceptors, which are pre-positioned due to the coordination by the Ba ions, and therefore the geometry of these inter­actions is slightly forced. Only bonding between the amide-NH and nitroso-O sites is favourable (N3⋯O1^vi^ = 3.084 (5) Å; symmetry code: (vi) −*x* + 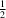, −*y* + 1, *z* + 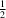; Table 2 ▸) and it may be regarded as a stabilizing factor for the present mutual orientation of the coordinated (ccnm)^−^ anions (Fig. 3 ▸*a*). At the same time, a second inter­action of the NH_2_ group is particularly weak with the N⋯O separation appreciably exceeding the sum of van der Waals radii of 3.07 Å, whereas two symmetry-identical bonds with bridging water mol­ecule are associated with far less favourable angles at the H atoms [O2*W*⋯O2^vii^ = 2.868 (4) Å; O2*W*—H⋯O2^vii^ = 112 (6)°; symmetry code: (vii) −*x* + 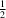, −*y*, *z* − 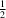] (Fig. 3 ▸*a*).

**Figure 5:**
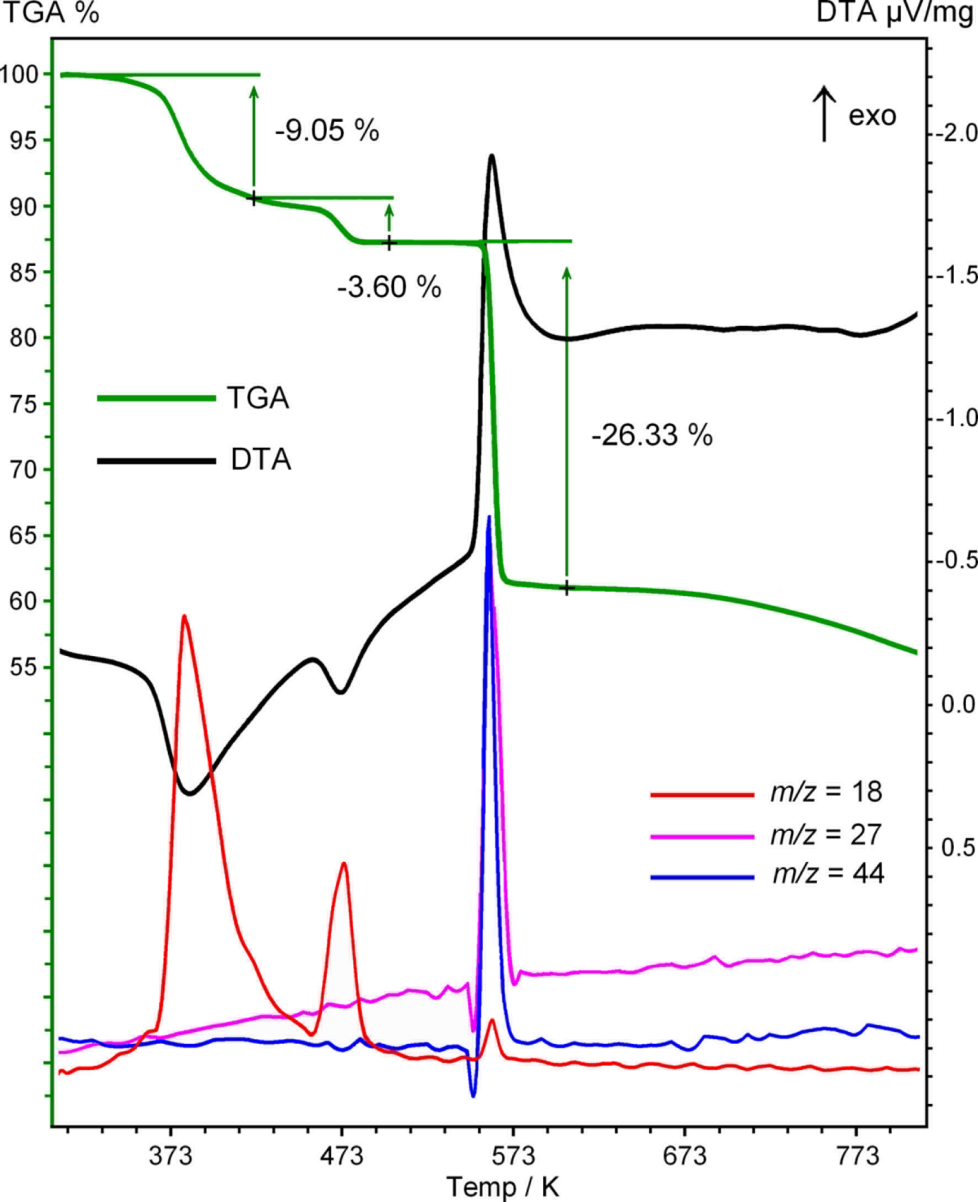
Combined TGA (green), DTA (black) and MS (red, blue, purple) plots for the title compound, in the temperature range 303–813 K (argon, heating rate 10 K min^−1^).

**Figure 6:**
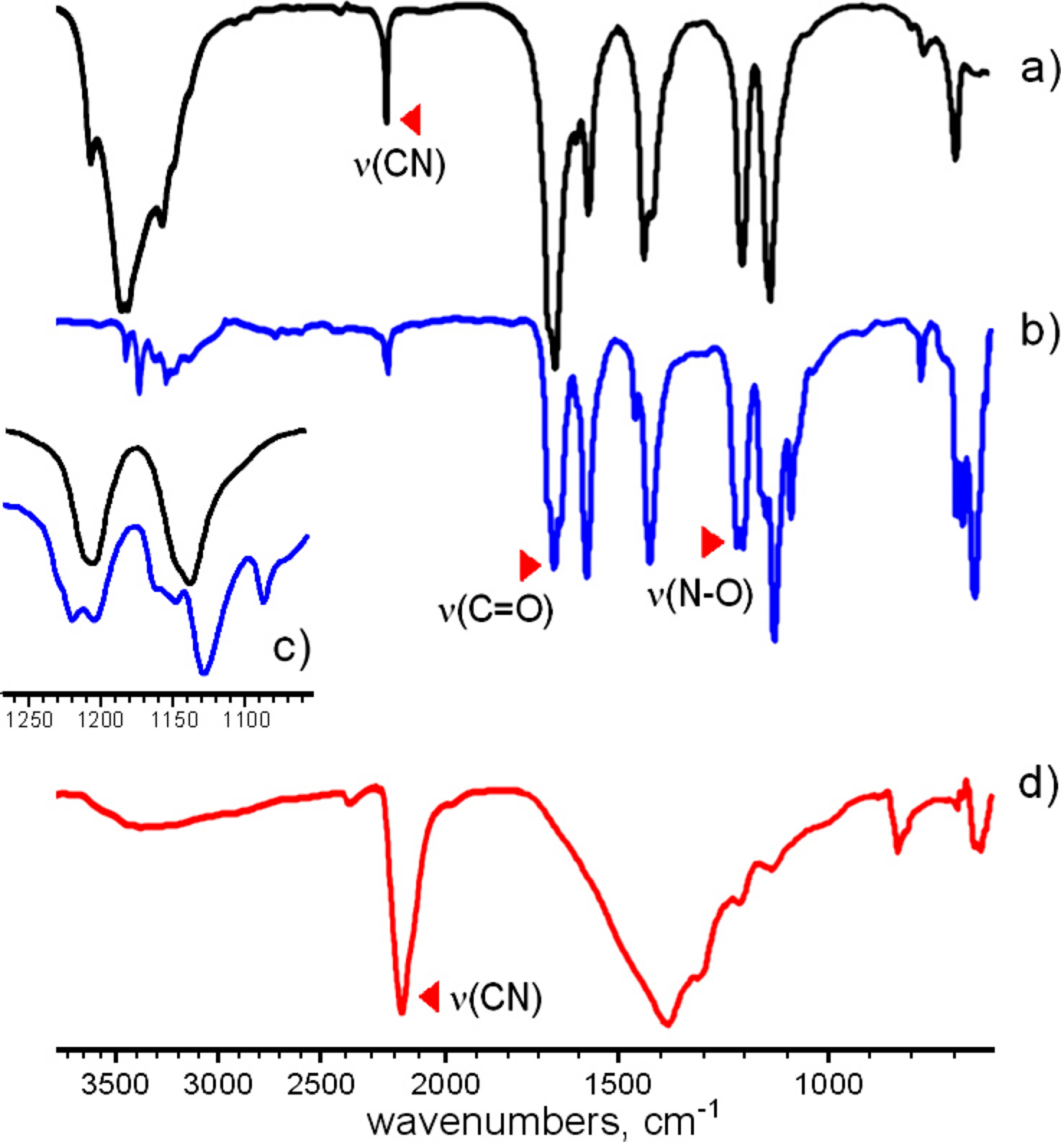
The IR spectra of (*a*) the title compound, (*b*) the product of its dehydration at 493 K for 2 h, (*c*) sections of the IR spectra, in the region of 1100–1250 cm^−1^, showing splitting of the ν(N—O) band upon dehydration, and (*d*) the final product of decomposition at 673 K.

The thermal properties of the title compound were examined by TG/DTA-MS analysis (Netzsch F1 Jupiter integrated with Aeolos mass spectrometer) (Fig. 5 ▸). There are two partially separated stages for endothermic weight losses in the temperature ranges of 333–423 K (−9.05 mass %) and 423–503 K (−3.60 mass %). These events are centred at 383 and 473 K, respectively. They are accompanied by peaks *m*/*z* = 18 and thus they correspond to the elimination of two and one water mol­ecules (− 2 H_2_O: calculated −8.66%; − H_2_O: calculated −4.33%). Therefore, the three types of water mol­ecules in the structure of **1** are only partially distinguishable by thermal analysis. The totally dehydrated material Ba(ccnm)_2_ is stable up to 558 K, when sharp exothermic decomposition occurs with the release of CO_2_ (*m*/*z* = 44), HCN (*m*/*z* = 27) and H_2_O (*m*/*z* = 18). For the attempted decomposition experiment in a small preparative scale of 100 mg, the sample exploded immediately after the temperature reached 558 K. The weight loss in the temperature range of 553–603 K is 26.33 mass % (− CO_2_, − 2 HCN, − H_2_O: calculated −27.92%). The resulting material remains intact to significantly higher temperatures, with only very slight outstretched weight loss observed above 693 K (Fig. 5 ▸). For comparison, the closely related Ba(ONC(CN)_2_)_2_·H_2_O decomposes at the comparable temperature of 536 K and also very violently (*‘to shatter the sample cups*’; Arulsamy *et al.*, 1999 ▸). However, the pathways of nitro­sodia­cyano­methanide degradation are different, since KONC(CN)_2_ forms (CN)_2_ and not HCN as in the present case (Jasim, 1989 ▸). The amount of the remaining brown amorphous material (60.00% to 723 K) suggests the composition of BaC_3_N_4_O (calculated: 59.04%). It cannot be attributed as Ba[NCO][N(CN)_2_], since either barium cyanate or di­cyano­amide themselves readily undergo thermal trimerization of the anions giving cyanurate C_3_N_3_O_3_^3−^, mixed anion cyanurates, tri­cyano­melaminates C_6_N_9_^3−^ and even larger condensation products. These species could be detected by a distinctive pattern in the FT–IR spectrum that reveals a very strong absorption band at 2165 cm^−1^ (Fig. 6 ▸). It corresponds to ν(C≡N) in the highly conjugated tri­cyano­melaminates observed, for example, in the spectrum of NaRb_5_(C_6_N_9_^3−^)_2_·4H_2_O [2164 cm^−1^; Reckeweg *et al.*, 2016 ▸], while a series of partially resolved bands in the 1060–1450 cm^−1^ region is well in accordance with the ring ν(C=N) for the mixture of C_3_N_3_O_3_^3−^ (Kalmutzki *et al.*, 2014 ▸) and C_6_N_9_^3−^ anions (Reckeweg *et al.*, 2016 ▸). There are no ν(C=O) or cyanate ν(C≡N) absorptions [2191 cm^−1^ for Ba_2_(C_3_N_3_O_3_^3−^)_2_(NCO); Tang *et al.*, 2019 ▸], suggesting a total conversion of the inter­mediate decomposition products.

Following the results of thermal analysis, the anhydrate Ba(ccnm)_2_ was prepared by calcination of the title compound at 493 K for 2 h. The FT–IR spectra of **1** and its dehydration product are very similar, beyond the elimination of broad absorption bands in the ν(O—H) region in the latter case. The strong ν(N—O) absorption at 1202 cm^−1^ in the spectra of **1** is almost identical to the value of 1212 cm^−1^ for the ammonium salt (Domasevitch *et al.*, 2021 ▸) and both these compounds manifest a certain low-frequency shift when compared to the spectra of NMe_4_(ccnm) (1253 cm^−1^; Izgorodina *et al.*, 2010 ▸). This reflects a perceptible sensitivity of ν(N—O) bands either to the effects of ion–dipole coordination for the alkaline earth metal salts or for relatively strong multiple hydrogen bonding observed for NH_4_(ccnm). It is not surprising that dehydration causes splitting of the ν(N—O) absorption (1206 and 1214 cm^−1^) (Fig. 6 ▸*c*). Since the blue shift of ν(N—O) is in line with a higher N—O bond order and also with stronger *M*—N coordination (Domasevitch *et al.*, 2021 ▸), one can suppose that upon elimination of the aqua ligands the metal–nitroso inter­action becomes stronger due to the involvement of the N atom. A realistic pattern could consider side-coordination of the N—O group with the formation of a three-membered chelate ring, similar to the structure of discrete complex anions [*Ln*(ccnm)_6_]^3−^ (Chesman *et al.*, 2010 ▸) and to the local [Ba(NO)_6_] motif in *N*,*N*′-di­methyl­iso­nitro­somalonamide (Raston & White, 1976 ▸). In the spectra of **1** and its anhydrate, ν(C≡N) is present as a medium-intense band at 2222 cm^−1^. It is characteristic for ionic salts of conjugated cyano­nitroso anions, whereas the neutral H(ccnm) derivatives exhibit only very weak ν(C≡N) absorption around 2236 cm^−1^.

In brief, the present system suggests small cyano­nitroso anions to be suitable building blocks for the construction of extended framework solids. The behaviour of the hard Lewis basic nitroso-O atoms towards alkaline earth cations may be considered predictable with regard to generating multiple *M*—O—*M* bridges and a subsequent face- or edge-sharing fusion of the coordination polyhedra. Such cyano­nitroso compounds may be involved as possible single-source precursors for the thermal solid-state metathesis reactions toward alkaline earth metal carbonitride materials.

## 4. Database survey

A search of the Cambridge Structural Database (CSD version 5.43, update of November 2022; Groom *et al.*, 2016 ▸) reveals no alkaline earth metal carbamoyl­cyano­nitro­somethanides, while a series of barium salts with different nitroso-anions accounts for twelve hits. The present nitroso-O—Ba linkage, which delivers chains of Ba_2_O_2_ rhombes sharing their Ba-vertices, is reminiscent of the polymeric motif adopted by the most closely related nitro­sodi­cyano­methanide (Arulsamy *et al.*, 1999 ▸). This structure is not deposited in the CSD. Triple nitroso-O bridging of Ba ions is also known as a local motif in *N*,*N*′-di­methyl­iso­nitro­somalonamide (refcode: MIMALB; Raston & White, 1976 ▸). The Ba⋯Ba separation for this [Ba(μ-ON)_3_Ba] fragment [4.2414 (7) Å] was slightly shorter than 4.4102 (7) Å for [Ba(μ-ON)_2_(μ-OH_2_)Ba] in the title structure. However, the supra­molecular patterns for Ba iso­nitro­somalonamide (refcode: INMALB; Raston & White, 1976 ▸) and also for the comparable *N*,*N*′-di­methyl­violurate (refcode: LEDYOM; Lorenz *et al.*, 2022 ▸) were essentially dominated by the formation of different *N*,*O*- and *O*,*O*-chelate fragments, which mitigate against the generation of high-dimensional frameworks. In this way, the dimensionality of Ba iso­nitro­somalonamide was decreased down to two (Raston & White, 1976 ▸), as may be compared with complex three-dimensional frameworks found for **1** and for Ba(ONC(CN)_2_)_2_·H_2_O (Arulsamy *et al.*, 1999 ▸).

## 5. Synthesis and crystallization

The carbamoyl­cyano­nitro­somethanide was prepared in 92% yield by a modified method for the nitro­sation of cyano­acetamide, with isolation of the reaction product in form of the silver salt Ag(ccnm) (Domasevitch *et al.*, 2024 ▸).

A solution of 4.20 g (50 mmol) of cyano­acetamide and 4.14 g (60 mmol) of NaNO_2_ in 50 ml of water was cooled to 278 K and then 4.00 ml (70 mmol) of CH_3_COOH were added dropwise for 3 h with stirring. The mixture was allowed to stand for 12 d at 278–283 K in a stoppered flask to complete precipitation of a faintly yellow voluminous deposit, which represents the sodium hydrogen salt NaH(ccnm)_2_. The latter was dissolved at room temperature by addition of 100 ml of water, after which a solution of 8.49 g (50 mmol) of AgNO_3_ in 30 ml of water was added with stirring. A yellow–orange precipitate of Ag(ccnm) was formed immediately. It was filtered, washed thoroughly with 30 ml portions of water and methanol and dried in air. The yield was 10.11 g (92%).

For preparation of the barium salt **1**, 1.385 g (6.3 mmol, excess 5%) of the finely powdered solid Ag(ccnm) was added to a solution of 0.733 g (3.0 mmol) of BaCl_2_·2H_2_O in 25 ml of water and the mixture was stirred for 4 h. A light-grey deposit of AgCl was filtered off and washed with 2 ml of water. The obtained bright-yellow solution was slowly evaporated to a volume of 3–4 ml, giving several large orange crystals of the product in a yield of 1.084 g (87%). The crystals were stable in air for 10–15 d, but eventually they became opaque and lost their crystallinity. Analysis (%) calculated for C_6_H_10_N_6_O_7_Ba: C 17.34, H 2.43, N 20.27; found: C 17.49, H 2.27, N 20.49. IR (KBr, cm^−1^): 652 *w*, 686 *m*, 762 *w*, 1136 *vs*, 1202 *s*, 1422 *m*, 1444 *m*, 1578 *m*, 1608 *w*, 1658 *vs*, 2222 *m*, 3284 *br*, 3450 *br*, 3628 *br*.

## 6. Refinement

Crystal data, data collection and structure refinement details are summarized in Table 3 ▸. All hydrogen atoms were located in difference maps and then refined with isotropic displacement parameters and with soft similarity restraints for the O—H bond lengths and H—O—H bond angles for water mol­ecules and N—H bond lengths and C—N—H bond angles for the amide group, which results in O—H = 0.84 (3)–0.85 (3) Å and N—H = 0.87 (3) Å. Two outliers (10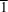 and 00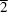) were omitted in the last cycles of refinement.

## Supplementary Material

Crystal structure: contains datablock(s) global, I. DOI: 10.1107/S2056989024008375/yz2057sup1.cif

Structure factors: contains datablock(s) I. DOI: 10.1107/S2056989024008375/yz2057Isup2.hkl

CCDC reference: 2379364

Additional supporting information:  crystallographic information; 3D view; checkCIF report

## Figures and Tables

**Table 1:** Selected bond lengths (Å)

Ba1—O2^i^	2.763 (3)	Ba1—O1^v^	2.845 (3)
Ba1—O2^ii^	2.763 (3)	Ba1—O1*W*	2.860 (5)
Ba1—O1^iii^	2.787 (3)	Ba1—O2*W*^iv^	2.951 (5)
Ba1—O1	2.787 (3)	Ba1—O2*W*	2.961 (4)
Ba1—O1^iv^	2.845 (3)		

Symmetry codes: (i) 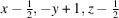; (ii) 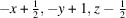; (iii) 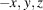; (iv) 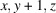; (v) 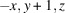.

**Table 2:** Hydrogen-bond geometry (Å, °)

*D*—H⋯*A*	*D*—H	H⋯*A*	*D*⋯*A*	*D*—H⋯*A*
N3—H1⋯O1^vi^	0.87 (3)	2.29 (5)	3.084 (5)	151 (5)
N3—H2⋯O1*W*	0.87 (3)	2.64 (3)	3.490 (4)	169 (5)
O1*W*—H1*W*⋯O3*W*	0.85 (3)	1.94 (3)	2.782 (7)	169 (9)
O1*W*—H2*W*⋯O3*W*^iv^	0.85 (3)	2.05 (4)	2.877 (7)	162 (7)
O2*W*—H3*W*⋯O2^vii^	0.85 (3)	2.44 (7)	2.868 (4)	112 (6)
O3*W*—H4*W*⋯N2^viii^	0.84 (3)	2.12 (4)	2.933 (5)	161 (5)

Symmetry codes: (iv) 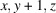; (vi) 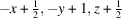; (vii) 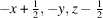; (viii) 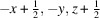.

**Table 3:** Experimental details

Crystal data	
Chemical formula	[Ba(C_3_H_2_N_3_O_2_)_2_(H_2_O)_2_]·H_2_O
*M*_r_	415.54
Crystal system, space group	Orthorhombic, *Pmn*2_1_
Temperature (K)	173
*a*, *b*, *c* (Å)	13.6667 (17), 4.4102 (7), 11.2816 (14)
*V* (Å^3^)	679.97 (16)
*Z*	2
Radiation type	Mo *K*α
μ (mm^−1^)	2.96
Crystal size (mm)	0.22 × 0.18 × 0.15

Data collection	
Diffractometer	Stoe Image plate diffraction system-2T
Absorption correction	Numerical [*X-RED* (Stoe & Cie, 2001 ▸) and *X-SHAPE* (Stoe & Cie, 1999 ▸)]
*T*_min_, *T*_max_	0.310, 0.399
No. of measured, independent and observed [*I* > 2σ(*I*)] reflections	3251, 1696, 1688
*R*_int_	0.016
(sin θ/λ)_max_ (Å^−1^)	0.692

Refinement	
*R*[*F*^2^ > 2σ(*F*^2^)], *wR*(*F*^2^), *S*	0.017, 0.043, 1.15
No. of reflections	1696
No. of parameters	119
No. of restraints	13
H-atom treatment	All H-atom parameters refined
Δρ_max_, Δρ_min_ (e Å^−3^)	0.72, −1.87
Absolute structure	Flack *x* determined using 668 quotients [(*I*^+^)−(*I*^−^)]/[(*I*^+^)+(*I*^−^)] (Parsons *et al.*, 2013 ▸)
Absolute structure parameter	−0.013 (14)

Computer programs: *X-AREA* (Stoe & Cie, 2016 ▸), *SHELXS97* (Sheldrick, 2008 ▸), *SHELXL2019/2* (Sheldrick, 2015 ▸), *DIAMOND* (Brandenburg, 1999 ▸) and *WinGX* (Farrugia, 2012 ▸).

## supplementary crystallographic information

### Poly[[µ-aqua-aquabis(µ3-carbamoylcyanonitrosomethanido)barium] monohydrate] Crystal data

**Table:**

[Ba(C_3_H_2_N_3_O_2_)_2_(H_2_O)_2_]·H_2_O	*D*_x_ = 2.030 Mg m^−^^3^
*M**_r_* = 415.54	Mo *K*α radiation, λ = 0.71073 Å
Orthorhombic, *P**m**n*2_1_	Cell parameters from 3251 reflections
*a* = 13.6667 (17) Å	θ = 4.6–29.4°
*b* = 4.4102 (7) Å	µ = 2.96 mm^−^^1^
*c* = 11.2816 (14) Å	*T* = 173 K
*V* = 679.97 (16) Å^3^	Prism, yellow
*Z* = 2	0.22 × 0.18 × 0.15 mm
*F*(000) = 400	

### Poly[[µ-aqua-aquabis(µ3-carbamoylcyanonitrosomethanido)barium] monohydrate] Data collection

**Table:**

Stoe Image plate diffraction system-2T diffractometer	1688 reflections with *I* > 2σ(*I*)
Radiation source: fine-focus sealed tube	*R*_int_ = 0.016
φ oscillation scans	θ_max_ = 29.4°, θ_min_ = 4.6°
Absorption correction: numerical [X-RED (Stoe & Cie, 2001) and X-SHAPE (Stoe & Cie, 1999)]	*h* = −15→18
*T*_min_ = 0.310, *T*_max_ = 0.399	*k* = −5→6
3251 measured reflections	*l* = −15→13
1696 independent reflections	

### Poly[[µ-aqua-aquabis(µ3-carbamoylcyanonitrosomethanido)barium] monohydrate] Refinement

**Table:**

Refinement on *F*^2^	Secondary atom site location: difference Fourier map
Least-squares matrix: full	Hydrogen site location: difference Fourier map
*R*[*F*^2^ > 2σ(*F*^2^)] = 0.017	All H-atom parameters refined
*wR*(*F*^2^) = 0.043	*w* = 1/[σ^2^(*F*_o_^2^) + (0.0318*P*)^2^] where *P* = (*F*_o_^2^ + 2*F*_c_^2^)/3
*S* = 1.15	(Δ/σ)_max_ < 0.001
1696 reflections	Δρ_max_ = 0.72 e Å^−^^3^
119 parameters	Δρ_min_ = −1.87 e Å^−^^3^
13 restraints	Absolute structure: Flack *x* determined using 668 quotients [(*I*^+^)-(*I*^-^)]/[(*I*^+^)+(*I*^-^)] (Parsons *et al.*, 2013)
Primary atom site location: structure-invariant direct methods	Absolute structure parameter: −0.013 (14)

### Poly[[µ-aqua-aquabis(µ3-carbamoylcyanonitrosomethanido)barium] monohydrate] Special details

**Table:**

Geometry. All esds (except the esd in the dihedral angle between two l.s. planes) are estimated using the full covariance matrix. The cell esds are taken into account individually in the estimation of esds in distances, angles and torsion angles; correlations between esds in cell parameters are only used when they are defined by crystal symmetry. An approximate (isotropic) treatment of cell esds is used for estimating esds involving l.s. planes.

### Poly[[µ-aqua-aquabis(µ3-carbamoylcyanonitrosomethanido)barium] monohydrate] Fractional atomic coordinates and isotropic or equivalent isotropic displacement parameters (Å^2^)

**Table:**

	*x*	*y*	*z*	*U*_iso_*/*U*_eq_	
Ba1	0.000000	0.71083 (4)	0.46009 (2)	0.01511 (8)	
O1	0.1246 (2)	0.2193 (6)	0.4964 (3)	0.0242 (6)	
O2	0.3561 (3)	0.2641 (7)	0.7884 (4)	0.0338 (7)	
N1	0.1548 (2)	0.3428 (7)	0.5958 (3)	0.0204 (6)	
N2	0.3448 (3)	−0.1379 (11)	0.5230 (4)	0.0392 (8)	
N3	0.2319 (3)	0.6036 (9)	0.7971 (3)	0.0277 (6)	
C1	0.2387 (3)	0.2447 (9)	0.6375 (4)	0.0191 (6)	
C2	0.2979 (2)	0.0271 (9)	0.5748 (3)	0.0237 (7)	
C3	0.2796 (2)	0.3730 (9)	0.7480 (3)	0.0216 (6)	
O1W	0.000000	0.8440 (11)	0.7082 (5)	0.0446 (12)	
O2W	0.000000	0.2093 (9)	0.2856 (4)	0.0284 (9)	
O3W	0.000000	0.3577 (11)	0.8653 (4)	0.0304 (8)	
H1	0.253 (5)	0.681 (12)	0.864 (4)	0.05 (2)*	
H2	0.178 (3)	0.666 (9)	0.765 (5)	0.027 (12)*	
H1W	0.000000	0.712 (13)	0.763 (7)	0.04 (3)*	
H2W	0.000000	1.019 (8)	0.740 (6)	0.04 (2)*	
H3W	0.0509 (10)	0.192 (17)	0.243 (5)	0.07 (3)*	
H4W	0.0508 (10)	0.334 (15)	0.906 (5)	0.048 (18)*	

### Poly[[µ-aqua-aquabis(µ3-carbamoylcyanonitrosomethanido)barium] monohydrate] Atomic displacement parameters (Å^2^)

**Table:**

	*U*^11^	*U*^22^	*U*^33^	*U*^12^	*U*^13^	*U*^23^
Ba1	0.01474 (10)	0.01439 (11)	0.01620 (11)	0.000	0.000	0.0010 (3)
O1	0.0220 (12)	0.0259 (12)	0.0248 (14)	−0.0015 (8)	−0.0088 (9)	0.0003 (9)
O2	0.0310 (16)	0.0345 (14)	0.0359 (19)	0.0029 (11)	−0.0197 (14)	−0.0041 (12)
N1	0.0186 (12)	0.0210 (13)	0.0217 (15)	−0.0023 (11)	−0.0030 (10)	0.0003 (11)
N2	0.0359 (18)	0.0426 (19)	0.039 (2)	0.0108 (17)	0.0031 (15)	−0.0062 (18)
N3	0.0306 (16)	0.0290 (18)	0.0234 (17)	0.0021 (15)	−0.0037 (12)	−0.0045 (14)
C1	0.0184 (15)	0.0190 (15)	0.0199 (18)	−0.0021 (12)	−0.0029 (12)	0.0032 (12)
C2	0.0208 (14)	0.0268 (16)	0.0235 (17)	−0.0006 (12)	−0.0046 (11)	−0.0005 (13)
C3	0.0211 (13)	0.0243 (15)	0.0195 (16)	−0.0054 (13)	−0.0043 (11)	0.0022 (13)
O1W	0.088 (4)	0.022 (2)	0.024 (2)	0.000	0.000	0.0013 (18)
O2W	0.026 (2)	0.031 (2)	0.028 (2)	0.000	0.000	−0.0024 (15)
O3W	0.0283 (18)	0.035 (2)	0.028 (2)	0.000	0.000	0.0081 (18)

### Poly[[µ-aqua-aquabis(µ3-carbamoylcyanonitrosomethanido)barium] monohydrate] Geometric parameters (Å, º)

**Table:**

Ba1—O2^i^	2.763 (3)	N2—C2	1.132 (5)
Ba1—O2^ii^	2.763 (3)	N3—C3	1.329 (5)
Ba1—O1^iii^	2.787 (3)	N3—H1	0.87 (3)
Ba1—O1	2.787 (3)	N3—H2	0.87 (3)
Ba1—O1^iv^	2.845 (3)	C1—C2	1.441 (5)
Ba1—O1^v^	2.845 (3)	C1—C3	1.478 (5)
Ba1—O1W	2.860 (5)	O1W—H1W	0.85 (3)
Ba1—O2W^iv^	2.951 (5)	O1W—H2W	0.85 (3)
Ba1—O2W	2.961 (4)	O2W—H3W	0.85 (3)
O1—N1	1.313 (4)	O2W—H3W^iii^	0.85 (3)
O2—C3	1.237 (5)	O3W—H4W	0.84 (3)
N1—C1	1.313 (5)	O3W—H4W^iii^	0.84 (3)
			
O2^i^—Ba1—O2^ii^	90.78 (19)	O1—Ba1—O2W	61.11 (9)
O2^i^—Ba1—O1^iii^	72.50 (10)	O1^iv^—Ba1—O2W	133.20 (8)
O2^ii^—Ba1—O1^iii^	124.70 (9)	O1^v^—Ba1—O2W	133.20 (8)
O2^i^—Ba1—O1	124.70 (9)	O1W—Ba1—O2W	143.53 (14)
O2^ii^—Ba1—O1	72.50 (10)	O2W^iv^—Ba1—O2W	96.48 (15)
O1^iii^—Ba1—O1	75.32 (12)	N1—O1—Ba1	89.72 (19)
O2^i^—Ba1—O1^iv^	119.71 (9)	N1—O1—Ba1^vi^	129.7 (2)
O2^ii^—Ba1—O1^iv^	69.11 (10)	Ba1—O1—Ba1^vi^	103.07 (9)
O1^iii^—Ba1—O1^iv^	163.22 (13)	C3—O2—Ba1^vii^	147.6 (3)
O1—Ba1—O1^iv^	103.07 (9)	C1—N1—O1	116.4 (3)
O2^i^—Ba1—O1^v^	69.11 (10)	C3—N3—H1	120 (3)
O2^ii^—Ba1—O1^v^	119.71 (8)	C3—N3—H2	119 (3)
O1^iii^—Ba1—O1^v^	103.07 (9)	H1—N3—H2	121 (5)
O1—Ba1—O1^v^	163.22 (13)	N1—C1—C2	122.2 (4)
O1^iv^—Ba1—O1^v^	73.52 (12)	N1—C1—C3	120.4 (3)
O2^i^—Ba1—O1W	132.68 (9)	C2—C1—C3	117.2 (3)
O2^ii^—Ba1—O1W	132.68 (9)	N2—C2—C1	178.0 (4)
O1^iii^—Ba1—O1W	90.90 (10)	O2—C3—N3	123.9 (4)
O1—Ba1—O1W	90.90 (10)	O2—C3—C1	118.8 (4)
O1^iv^—Ba1—O1W	72.36 (10)	N3—C3—C1	117.3 (3)
O1^v^—Ba1—O1W	72.36 (10)	Ba1—O1W—H1W	125 (6)
O2^i^—Ba1—O2W^iv^	60.16 (8)	Ba1—O1W—H2W	127 (5)
O2^ii^—Ba1—O2W^iv^	60.16 (8)	H1W—O1W—H2W	108 (6)
O1^iii^—Ba1—O2W^iv^	132.66 (8)	Ba1^vi^—O2W—Ba1	96.48 (15)
O1—Ba1—O2W^iv^	132.66 (8)	Ba1^vi^—O2W—H3W	108 (5)
O1^iv^—Ba1—O2W^iv^	60.60 (9)	Ba1—O2W—H3W	116 (4)
O1^v^—Ba1—O2W^iv^	60.60 (9)	Ba1^vi^—O2W—H3W^iii^	108 (5)
O1W—Ba1—O2W^iv^	120.00 (13)	Ba1—O2W—H3W^iii^	116 (4)
O2^i^—Ba1—O2W	64.14 (9)	H3W—O2W—H3W^iii^	110 (7)
O2^ii^—Ba1—O2W	64.14 (9)	H4W—O3W—H4W^iii^	111 (6)
O1^iii^—Ba1—O2W	61.11 (9)		
			
Ba1—O1—N1—C1	−160.6 (3)	Ba1^vii^—O2—C3—C1	176.7 (4)
Ba1^vi^—O1—N1—C1	92.6 (4)	N1—C1—C3—O2	176.0 (4)
O1—N1—C1—C2	3.7 (6)	C2—C1—C3—O2	−8.3 (6)
O1—N1—C1—C3	179.1 (3)	N1—C1—C3—N3	−4.9 (5)
Ba1^vii^—O2—C3—N3	−2.3 (8)	C2—C1—C3—N3	170.8 (4)

Symmetry codes: (i) *x*−1/2, −*y*+1, *z*−1/2; (ii) −*x*+1/2, −*y*+1, *z*−1/2; (iii) −*x*, *y*, *z*; (iv) *x*, *y*+1, *z*; (v) −*x*, *y*+1, *z*; (vi) *x*, *y*−1, *z*; (vii) −*x*+1/2, −*y*+1, *z*+1/2.

### Poly[[µ-aqua-aquabis(µ3-carbamoylcyanonitrosomethanido)barium] monohydrate] Hydrogen-bond geometry (Å, º)

**Table:**

*D*—H···*A*	*D*—H	H···*A*	*D*···*A*	*D*—H···*A*
N3—H1···O1^vii^	0.87 (3)	2.29 (5)	3.084 (5)	151 (5)
N3—H2···O1*W*	0.87 (3)	2.64 (3)	3.490 (4)	169 (5)
O1*W*—H1*W*···O3*W*	0.85 (3)	1.94 (3)	2.782 (7)	169 (9)
O1*W*—H2*W*···O3*W*^iv^	0.85 (3)	2.05 (4)	2.877 (7)	162 (7)
O2*W*—H3*W*···O2^viii^	0.85 (3)	2.44 (7)	2.868 (4)	112 (6)
O3*W*—H4*W*···N2^ix^	0.84 (3)	2.12 (4)	2.933 (5)	161 (5)

Symmetry codes: (iv) *x*, *y*+1, *z*; (vii) −*x*+1/2, −*y*+1, *z*+1/2; (viii) −*x*+1/2, −*y*, *z*−1/2; (ix) −*x*+1/2, −*y*, *z*+1/2.
